# Upregulated MicroRNA-25 Mediates the Migration of Melanoma Cells by Targeting DKK3 through the WNT/β-Catenin Pathway

**DOI:** 10.3390/ijms17111124

**Published:** 2016-10-27

**Authors:** Jia Huo, Yanfei Zhang, Ruilian Li, Yuan Wang, Jiawen Wu, Dingwei Zhang

**Affiliations:** Department of Dermatology, the Second Hospital of Xi’an Jiaotong University, Xi’an 710004, China; huojjaa@126.com (J.H.); chtu201108@126.com (Y.Z.); shwang153@126.com (R.L.); megy@163.com (Y.W.); jiawen702@126.com (J.W.)

**Keywords:** miR-25, DKK3, melanoma

## Abstract

Previous research indicates that microRNA-25 (miR-25) regulates carcinogenesis and the progression of various cancers, but the role of miR-25 in melanoma remains unclear. We observed that miR-25 was significantly upregulated in melanoma cell lines and tissue samples. Downregulation of miR-25 markedly suppressed invasion and proliferation of melanoma cells in vitro; however, overexpression of miR-25 markedly increased melanoma cell invasion and proliferation. Moreover, we observed Dickkopf-related protein 3 (DKK3) as a direct target of miR-25 in vitro. Upregulation of DKK3 partially attenuated the oncogenic effect of miR-25 on melanoma cells. Ectopic expression of miR-25 in melanoma cells induced β-catenin accumulation in nuclear and inhibited TCF4 (T cell factor 4) activity, as well as the expression of c-Myc and Cyclin D1. In a nude xenograft model, miR-25 upregulation significantly increased A375 melanoma growth. In summary, miR-25 is upregulated in melanoma and promotes melanoma cell proliferation and invasion, partially by targeting DKK3. These results were indicated that miR-25 may serve as a potential target for the treatment of melanoma in the future.

## 1. Introduction

Melanoma is one of the most severe forms of skin diseases, which is one of the most invasive, therapy-resistant, and metastatic tumors, with a five-year survival rate after diagnosis of only about 10% [1]. Melanoma, in the early stage, could be treated by resection of surgery, while melanoma in the late stage is difficult to treat with current therapies. For improving melanoma patients’ treatment, significant effort has been focused on elucidating the molecular and cellular mechanisms of this disease [2]. To date, the melanoma initiation and progression have been associated with signaling pathways, including PTEN (Phosphatase And Tensin Homolog)/AKT (V-Akt Murine Thymoma Viral Oncogene Homolog), WNT (Wingless-Type MMTV Integration Site Family)/β-catenin, and Ras/Raf/MEK (MAP kinse-ERK kinase)/ERK (extracellular regulated MAP kinase), and microRNAs (miRNAs) have been implicated as key regulators of these signaling pathways in recent studies [3,4].

MiRNAs, are a class of small, endogenous, noncoding RNAs with the length of ~22 nucleotides, which could induce post-transcriptional silencing of target sequence by interacting with their 3′-untranslated region (3′-UTR) of specific transcripts, resulting in translational repression and inducing gene silencing [5,6,7,8]. Mounting evidence in recent years has shown that miRNAs regulates many important biological processes, such as tumorigenesis, inflammation, and development.

Considering the above findings, we investigated the possible involvement of miRNAs in the expansion and functions of melanoma. We analyzed that at high expression levels, microRNA-25 (miR-25) played a critical role in regulating WNT pathway activity in melanoma to promote tumor progression. miR-25 activated the WNT pathway by targeting DKK3, thus not only enhancing the ability of melanoma to infiltrate into tumor tissue but also facilitating tumor invasion and metastasis by upregulating TCF4, c-Myc, and Cyclin D1 in vitro and in vivo. These results provide the first evidence of the molecular mechanisms that regulate the functions of tumor-associated melanoma and into the exploration of powerful targets for therapeutic strategy.

## 2. Results

### 2.1. Upregulation of miR-25 Is Associated with Melanoma

**Figure 1:**
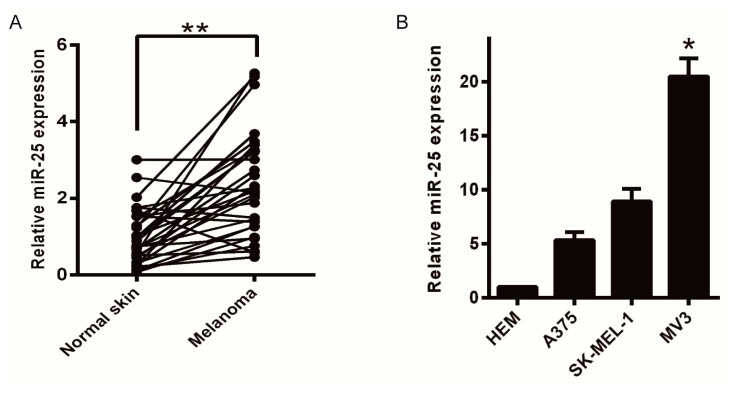
The expression of miR-25 is upregulated in melanoma tissues and melanoma cell lines. (**A**) The miR-25 expression level in melanoma tissue and associated normal tissues was measured by qRT-PCR (quantitative real-time polymerase chain reaction); (**B**) the expression of miR-25 in melanoma cell lines and the normal epithelial HEM (human epidermal melanocyte) cell line was measured by qRT-PCR. Data are expressed as the mean ± SD of three independent experiments. * *p* < 0.05, ** *p* < 0.01.

To measure the level of miR-25 expression in melanoma in clinic, quantitative real-time polymerase chain reaction (qRT-PCR) analysis was examined using 30 pairs of snap-frozen human primary melanoma and associated adjacent skin. As shown in Figure 1A, the expression of miR-25 in melanoma tissues was dramatically higher than that in pair-matched adjacent skin (*p* < 0.001). These results suggested that the upregulation of miR-25 was associated with the progression of melanoma. Then, we tested whether miR-25 expression was associated with malignant melanoma cells lines. Four selected cell lines: HEM (human epidermal melanocyte), MV3, SK-MEL-1, and A375, were compared of the miR-25 expression. Finally, miR-25 was upregulated to a high level in the malignant melanoma cell lines compared with the HEMs (Figure 1B). In conclusion, these results found that overexpression of miR-25 was markedly corresponding with the proliferation of malignant melanoma.

### 2.2. Downregulation of miR-25 Inhibits Melanoma Cell Proliferation and Motility

**Figure 2:**
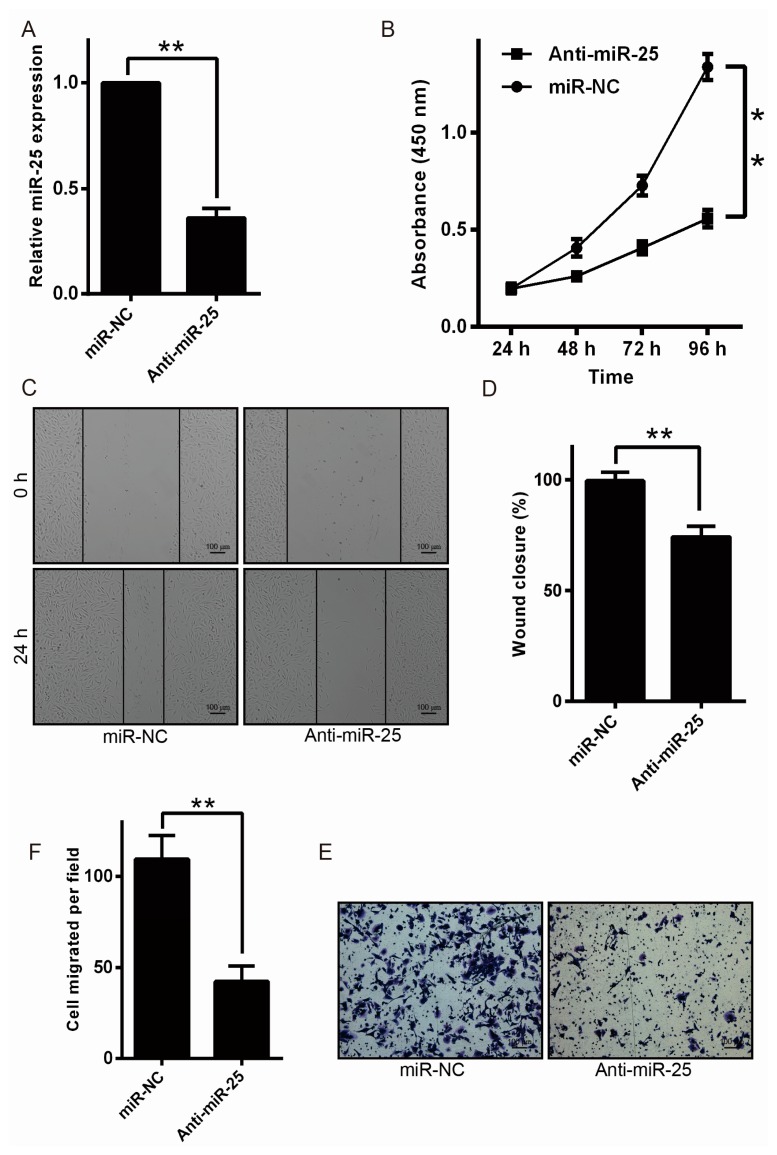
miR-25 downregulation inhibits melanoma cell motility and proliferation. (**A**) Anti-miR-25, or miR-NC, was transfected into MV3 cells and the levels of miR-25 were measured by qRT-PCR; (**B**) MV3 cells proliferation was evaluated using the MTT (Methyl Thiazolyl Tetrazolium); (**C**,**D**) MV3 cells was determined by wound-healing assays to detect the cell motility; (**E**,**F**) cell migration was detected using the transwell migration method. Data are expressed as the mean ± SD of three independent experiments. * *p* < 0.05, ** *p* < 0.01. Scale bar = 100 μm.

To detect the cell proliferation and motility of melanoma influencing by miR-25, anti-miR-25 (miR-25 inhibitor), or miR-NC, was transformed into MV3 cells. miR-25 expression was detected by qRT-PCR after transfection (Figure 2A). The MTT (Methyl Thiazolyl Tetrazolium) experiment showed that inhibition of miR-25 markedly inhibited melanoma cell proliferation in Figure 2B. The motility of melanoma cells was then detected using a wound-healing assay and transwell assays. The results showed that downregulation of miR-25 significantly inhibited melanoma cell migration in Figure 2C–F. These data revealed that miR-25 downregulation suppressed melanoma cell motility and proliferation in vitro.

### 2.3. miR-25 Targets DKK3 (Dickkopf-Related Protein 3)

**Figure 3:**
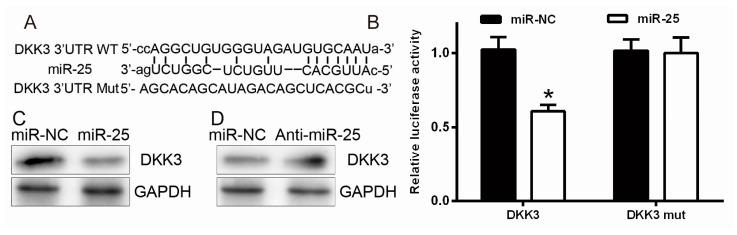
Prediction and identification of the miR-25 target. (**A**) Diagram for construction of the miR-25 binding site in the pGL3 vector. Mutation analysis of the miR-25 binding site; (**B**) identification of the miR-25 target gene. HEK293 cells were co-transfected with miR-NC, miR-25, and DKK3-pGL3 for the dual-luciferase assay. Renilla luciferase was expressed in pRL-TK vector, and was co-transfected with 3′-UTR of DKK3 for normalization; (**C**,**D**) DKK3 protein levels in miR-25 or anti-miR-25-treated HEK293 cells were measured by Western blot analysis. GAPDH used as an internal standard. * *p* < 0.05.

To further find the regulation mechanism by which miR-25 effected cell proliferation and migration, three target prediction programs with different algorithms: PicTar, TargetScan, and Miranda were screened for potential targets of miR-25. Upon the representation of the miR-25 sites in their 3′-UTRs, more than 100 mRNAs were candidates to be regulated by miR-25. The predicated targets with more than two-fold changes in expression were predicted of the candidates. Among these candidates, six genes (*DKK3* (*Dickkopf-related protein 3*), *DCAF6* (*DDB1 and CUL4 associated factor 6*), *FBXW7* (*F-box and WD repeat domain containing 7*), *FBXO11* (*F-box protein 11*), *MYO1B* (*myosin IB*), and *MAP2K4* (*mitogen-activated protein kinase kinase 4*)) were involved in the suppression of cancer metastasis. DKK3, a tumor suppressor in human tumorigenesis, was the most downregulated among all miR-25 target genes (Figure 3A). To confirm this finding, we subcloned the 3′-UTR mRNA region of DKK3, containing the predicted miR-25 site (this is the wild type, WT) or the mutated site (mutant type, Mut), into the plasmids containing luciferase reporter gene. A dual-luciferase reporter system was used. Our data indicated that miR-25 overexpression obviously inhibited WT but not 3′-UTR (Mut) of DKK3 (Figure 3B). Furthermore, the Western blot showed that inhibition of miR-25 dramatically upregulated the protein level of DKK3, whereas miR-25 overexpression had the opposite effect (Figure 3C,D). In summary, these data indicated that miR-25 decreased DKK3 level by directly targeting its 3′-UTR.

### 2.4. Upregulation of DKK3 Partially Attenuates the Oncogenic of miR-25

**Figure 4:**
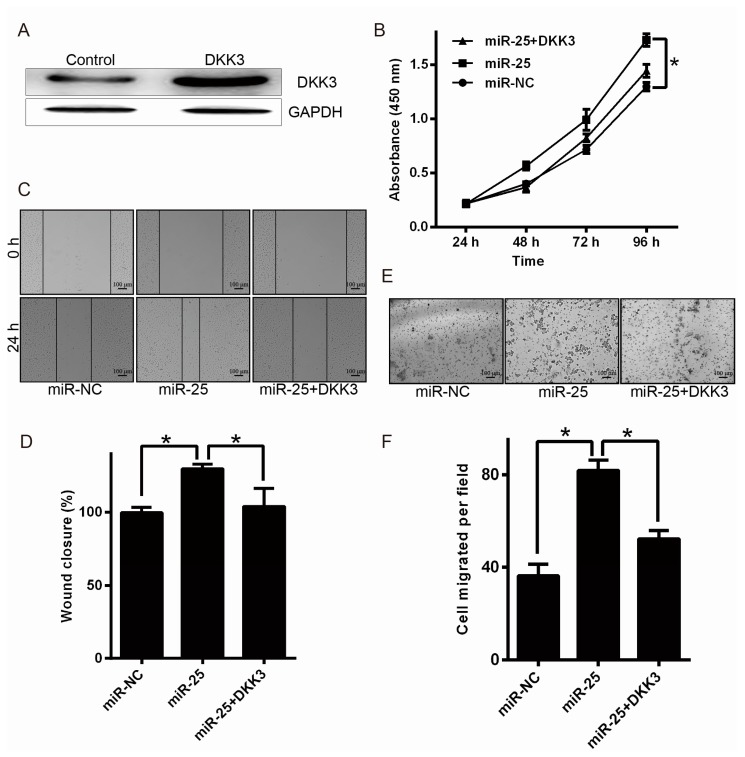
Overexpression of DKK3 attenuates the oncogenic influence of miR-25 in part. (**A**) MV3 were transfected with pcDNA3.1-DKK3 or control vector, and the protein levels of DKK3 were detected by Western blot analysis; (**B**) MV3 were co-transfected with miR-25 and pcDNA3.1-DKK3 vector or control, and the proliferation of MV3 was tested by the MTT assay; (**C**,**D**) MV3 motility of pretreated cells were measured by wound-healing assays; (**E**,**F**) pretreated MV3 motility was measured by transwell assays. Data are expressed as the mean ± SD of three independent experiments. * *p* < 0.05. Scale bar = 100 μm.

To explore whether DKK3 overexpression could attenuate the effect of miR-25 on melanoma cells, pcDNA3.1-DKK3 plasmid or the control vector was transfected into MV3. The influence of DKK3 was identified by Western blot analysis (Figure 4A). The MTT (Figure 4B), wound-healing, and transwell migration assays (Figure 4C–F) indicated that DKK3 supplementation could dramatically attenuate the effect of miR-25 on melanoma cells. Our findings suggest that miR-25 promotes melanoma cell proliferation and motility partially by targeting DKK3.

### 2.5. Overexpreesion of miR-25 Promotes Cell Proliferation and Induces β-Catenin/TCF4 Signaling Activation

**Figure 5:**
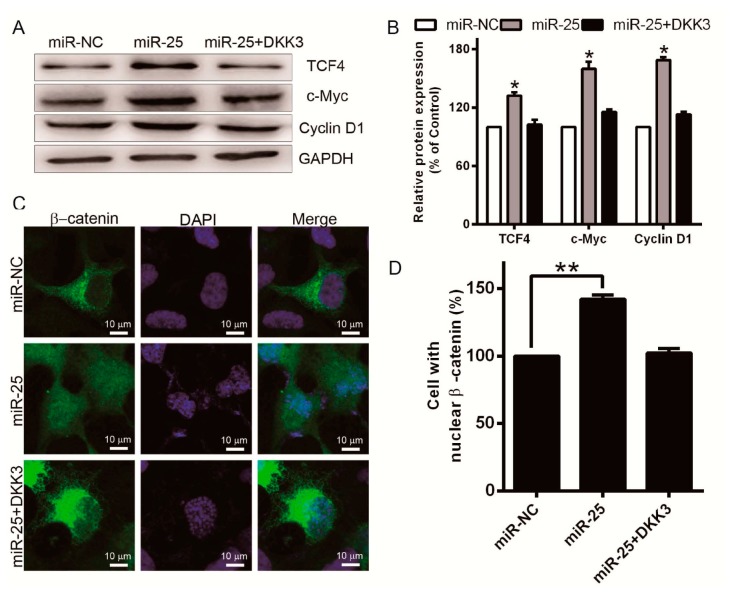
miR-25 inhibits nuclear translocation of β-catenin, and expression of TCF4 targets Cyclin D1 and c-Myc. (**A**) TCF4, Cyclin D1, and c-Myc level at the indicated points after miR-25 or miR-25, plus pcDNA-DKK3 transfection in MV3 cells was measured by Western blot. The level of GAPDH was emplored as the loading standard; (**B**) the protein levels were analyzed using the Quantity One software (Bio-Rad Laboratory, Inc., Hercules, CA, USA). The black histogram represents the optical densities of the signals quantified by densitometric analysis and represented as TCF4, c-Myc, or Cyclin D1 intensity/GAPDH to normalize for loading control; (**C**,**D**) MV3 were transfected with miR-NC, miR-25, or miR-25 plus pCDNA-DKK3. Localization of β-catenin was analyzed by immunofluorescence 48 h after transfection. 4′,6-Diamidino-2-phenylindole (DAPI) (blue) was used for nuclei staining. Scale bar = 10 μm. * *p* < 0.05, ** *p* < 0.01.

To explore the function of miR-25 in promoting melanoma cell proliferation, miR-25 was overexpressed in MV3 cells. We found that the protein levels of TCF4 (T cell factor 4), c-Myc, and Cyclin D1 were dramatically upregulated in cells with miR-25 transfection compared with control transfection (Figure 5A,B). In contrast, co-transfection of miR-25 and pCDNA-DKK3 resulted in opposite effects on the expression of c-Myc, Cyclin D1, and TCF4. We analyzed the localization of β-catenin by immunofluorescence suggested that the cells with nuclear β-catenin also upregulated by overexpression of miR-25 (Figure 5C,D). Then, nuclear localization of β-catenin was decreased in response to pCDNA-DKK3 transfection (Figure 5C,D), suggesting that miR-25 upregulation is sufficient to induce cell proliferation and β-catenin/TCF4 pathway, at least in some melanoma cells.

### 2.6. miR-25 Promotes Melanoma Tumor Growth in Nude Mice

**Figure 6:**
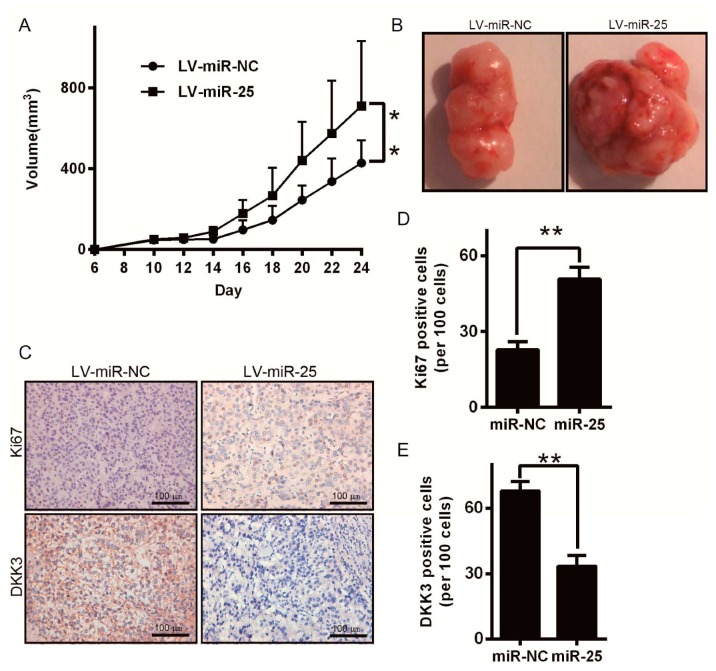
(**A**) A375 cells infected with LV-miR-25 or LV-miR-NC lentivirus were injected subcutaneously into nude mice. From day 6, the tumor sizes were analyzed every other day, and tumor growth curves were measured. Data are presented as the mean ± SD tumor volume; (**B**) twenty-four days after injection of A375 cells, the mice were sacrificed and photographed. Scale bar = 100 μm; (**C**) immunohistochemical (IHC) staining of Ki67 and DKK3 was performed using serial sections of subcutaneous xenograft tumors; scale bar = 100 μm; (**D**,**E**) Ki67 and DKK3 protein levels were measured by IHC in tissue sections from (**C**). ** *p* < 0.01.

As the important functions of miR-25 in melanoma, we next used melanoma xenograft models to further confirm the above findings in vivo. Since miR-25 was significantly upregulated in melanoma, we constructed a recombinant lentiviral vector named LV-miR-25 (and LV-miR-NC as control) to infect A375 cells (Figure S1A) and the upregulation of miR-25 (Figure S1B). The LV-miR-25 or LV-miR-NC-infected A375 cells were then injected into the flanks of a thymic nude mouse to establish subcutaneous melanoma xenografts. Twenty-four days after injection, the tumors were removed and measured. The tumor sizes in the LV-miR-25 group were much larger than those in the LV-miR-NC group (Figure 6A,B). To find out the mechanisms underlying miR-25-mediated tumor growth, resected tissues from the subcutaneous xenograft tumors were measured to identify the expression of Ki67 and DKK3. As shown in Figure 6C–E, the LV-miR-25 group displayed reduced DKK3 and Ki67 expression in the tumor tissues. Consistently, the LV-miR-25 group showed enhanced proliferation compared with the LV-miR-NC group (Figure 6C–E). These data indicated that miR-25 can facilitate melanoma growth in vivo.

## 3. Discussion

MicroRNAs play an important function in the tumor growth and formation [9]. Accumulating evidence indicates that dysregulation of miRNAs is frequently observed in multiple types of cancers and plays fundamental roles in tumor initiation and progression. miRNAs impact the dynamic balance between anti-tumor genes and oncogenes by regulating target genes, then influenced cancer growth [10]. miR-25 has been shown to be related with tumor carcinogenesis, including hepatocellular carcinoma [11], breast cancer [12], cholangiocarcinoma [13], and ovarian cancer [14]. However, the function of miR-25 in melanoma invasion and metastasis remains unclear.

In this work, we showed that miR-25 was significantly upregulated in melanoma compared with normal skin cells. Then, we used a cell model for further investigating miR-25 in melanoma. Then, we hypothesized that miR-25 downregulation could inhibit migration, indicating a potential strategy for the treatment of melanoma. Our in vitro work indicated that the miR-25 inhibitor dramatically inhibited migration and decreased cell proliferation. These functional studies suggested that miR-25 played a crucial role in tumor migration and, when dysregulated, might contribute to the inhibition of melanoma development.

Previous experiments have suggested that miR-25 level is significantly upregulated in some types of cancers, such as small cell lung cancer (SCLC), gastric cancer, and liver cancer [15,16,17]. Zhou and colleagues found that miR-25 was upregulated in both SCLC cells and human SCLC tissues. Downregulation of miR-25 dramatically decreased cancer cell proliferation, invasive capability, and resistance to cisplatin [15]. Additionally, Li and colleagues found that miR-25 was upregulated in plasma and primary tissues of gastric cancer (GC) patients with the feature of tumor node metastasis, lymph node metastasis or at stage III or IV [16]. Inhibition of miR-25 obviously reduced the invasion, proliferation, and metastasis of GC cells in vitro, and decreased their ability to develop distal pulmonary metastases and to undergo peritoneal dissemination in vivo. However, some investigators observed an opposite pattern of miR-25 expression in other cancers. For example, Zhou found that high expression of miR-25 resulted in the inhibition of colony formation in colorectal cancer [18]. It has been reported that miR-25 overexpression leads to a decrease in ANGPTL2 mRNA and protein level, whereas an adverse effect is observed when miR-25 is downregulated. In addition, it is demonstrated that miR-25 is overexpressed in most human cancers and has been widely studied in tissue or plasma, indicating that it can be used as a potential biomarker for cancer; yet the prognostic value of miR-25 in cancer remains unknown. Therefore, we conducted the present experiment to answer this question regarding the relationship between miR-25 expression and melanoma.

The Dickkopf family comprises four evolutionarily conserved members (DKK 1–4) and DKKL1, or soggy. Unlike others, it has previously been indicated that DKK3 does not influence WNT signaling [19]. The expression of DKK3 is low in malignant melanoma, and DKK-3-transfected cells revealed that DKK3 upregulated cell-cell adhesion and downregulated cell migration [20]. As detected by antagonism of cytoplasmic β-catenin, recently DKK3 was shown to display WNT inhibitory in the Saos-2 cell line [21]. Overexpression of DKK3 decreased TCF-driven gene activity, inhibited nuclear accumulation of β-catenin, and downregulated the WNT target genes *MYC* (*c*-*Myc*) and *CCND1* (*Cyclin* *D1*) expression in NSCLC cell lines [22]. DKK3 downregulated by shRNA (short hairpin RNA) led to an obvious increase in β-catenin/TCF-dependent gene activity in breast cancer cells [23].

Dysregulation of the WNT/β-catenin signaling pathway has been observed in various cancers. It has recently been shown that altered β-catenin expression is related with a poor prognosis in melanoma persons [24]. DKK3 has been shown to be inhibited by miR-25 in melanoma in our study. Thus, we concluded that miR-25 function as the WNT/β-catenin pathway regulator. This work indicated that miR-25 overexpression downregulated DKK3 and upregulated the level of β-catenin in the nucleus (Figure 5C), which indicated that miR-25 modulated β-catenin location via DKK3 in these cell lines. To identify whether miR-25 could regulate the WNT/β-catenin pathway, we detected the level of the downstream genes *TCF4*, *c-Myc* and *Cyclin* *D1* by Western blot. The data suggested that miR-25 and DKK3 overexpression could regulate the expression of these genes in melanoma. The protein level of c-Myc, which is not only a downstream gene of the WNT/β-catenin signaling pathway, but is also a cell motility gene, and was regulated by miR-25. This work indicated that miR-25 could modulate the genes downstream of the WNT/β-catenin pathway. Moreover, it could regulate cell motility. We indicated that WNT/β-catenin signaling activated miR-25 to promote migration and enhance motility of melanoma cells. The level of DKK3 was also negatively correlated with β-catenin expression. Therefore, our study revealed a novel mechanism by which WNT/β-catenin pathway contributes to cancer metastasis.

## 4. Materials and Methods

### 4.1. Cell Culture and Reagents

HEM cell lines, MV3, SK-HEP-1, and A375 were purchased from the American Type Culture Collection (ATCC, Rockville, MD, USA) and these cell lines were cultured in RPMI-1640 supplemented with 10% fetal bovine serum (FBS) (HyClone, Logan, UT, USA) and 1% penicillin/streptomycin (Invitrogen, Carlsbad, CA, USA) in a humidified incubator at 37 °C in a 5% CO_2_ atmosphere.

### 4.2. Tissue Samples

A total of 30 pairs of human primary melanoma tissues and related non-tumorous skin specimens were tested from The Second Affilated Hospital of Xi’an Jiaotong University, China. These melanoma cases were from 21 males and nine females, and none of the patients had received radiotherapy or chemotherapy before surgery. For research use, prior consent from patients and approval from the Ethics Committee of The Second Affiliated Hospital of Xi’an Jiaotong University were obtained. The patients provided informed consent. Patient anonymity has been preserved. All specimens had a confirmed pathological diagnosis and were classified according to World Health Organization (WHO) criteria.

### 4.3. RNA Isolation, Reverse Transcription and Quantitative Real-Time PCR (qRT-PCR)

Total RNA was isolated from the melanoma tissues and related non-tumorous skin tissues using TRIzol (Invitrogen, Carlsbad, CA, USA). Reverse-transcribed (RT) complementary DNA was reacted using the PrimeScript RT reagent Kit (TaKaRa, Dalian, China), and qRT-PCR was performed using SYBR Premix ExTaq (TaKaRa, Dalian, China) with the Stratagene Mx3000P real-time PCR system (Agilent Technologies, Inc., Santa Clara, CA, USA). The core sequence of miR-25 is 5′-AGTCTGGCTCTGTTCACGTTAC-3′. The primer sequence of miR-25 for the real-time PCR assay is that 5′-AGTCTGGCTCTGTTCACGTTA-3′. A common universal reverse primer and U6 was used as an endogenous standard. The relative level of miR-25 in each paired tumor and non-tumorous tissue was calculated by the 2^−ΔΔ*C*t^ method. For the mRNA analysis, cDNA was synthesized using reverse transcriptase (RT) kit (Promega, Madison, WI, USA). qRT-PCR was tested with SYBR Premix ExTaq kit with the TaKaRa SYBR real-time PCR system. GAPDH was used as an internal standard for quantification. 100 ng of the cDNA producted were quantified in a 20 μL using the following amplification program: 95 °C for 5 min, then by 40 amplification cycles of denaturation at 95 °C for 10 s, annealing at 61 °C for 20 s, and elongation at 72 °C for 10 s. Finally, a melting curve was generated by taking fluorescent measurements every 0.5 °C for 25 s from 50 °C to 95 °C. The relative level of mRNA was calculated by the 2^−ΔΔ*C*t^ method. All of the PCR reactions were repeated three times. Independent experiments were performed in triplicate.

### 4.4. Analysis of 3-(4,5-Dimethyl-2-thiazolyl)-2,5-diphenyl-2H-tetrazolium bromide (MTT)

Cells with miR-NC, miR-25 or miR25 + DKK3 transfection for 24 h, were plated (5000 cells per well) in 96-well plates and grown for 24, 48, 72, and 96 h (final miRNA concentration of 100 nM) in normal culture conditions. Cells were stained at the indicated time with 100 μL of sterile MTT dye (0.5 mg/mL, Sigma, St. Louis, MO, USA) for 4 h at 37 °C, then, removal of the culture medium and addition of 150 μL of dimethyl sulfoxide (DMSO) (Sigma). The absorbance was detected at 450 nm. All experiments were performed in triplicate.

### 4.5. Cell Migration Assays

Cell migration was performed according to a previous study. MV3 cells were transfected with miR-NC, miR-25 or miR25 + DKK3 for 24 h. Then, the MV3 cells in 100 μL of medium without FBS were seeded on a fibronectin-coated polycarbonate membrane insert in a transwell plate (Costar, Corning, NY, USA). 500 μL of medium supplemented with 10% FBS was added in the lower chamber as a chemoattractant. The cells were incubated for 6 h at 37 °C in a 5% CO_2_ atmosphere, the insert was washed with phosphate-buffered saline (PBS), and the cells on the top surface of the insert were removed softly with a cotton swab. Cells were fixed with methanol, stained with the indicated crystal violet solution, and counted in five predetermined fields (100×) under a microscope. All experiments were independently repeated at least three times.

### 4.6. Wound-Healing Assay

MV3 cells (1 × 10^6^ cells per well) were plated in six-well plates. The cells were transfected with miR-25 or anti-miR-25 after 24 h. The MV3 cells were wounded in serum-free medium containing 1% bovine serum albumin (BSA) with a sterile 200-μL pipette tip to remove cells after 24 h transfection. The progress of migration was taken photo in six regions, and for two days after wounding (0–24 h) using an inverted microscope (Nikon TMS-F, 301655, Tokyo, Japan) captured with a digital camera (Nikon Digital shot DS-L1, Tokyo, Japan). The migration of cells is calculated as the migration rate: (original scratch width—new scratch width)/original scratch width × 100%. Rescue experiment is also done by transfection of miR-NC, miR-25 or miR25 + DKK3 for 24 h in six-well plates. Wound-healing assays were performed according to the above procedure.

### 4.7. Transient Transfection with miR-25 or miRNA Inhibitors

miR-25 and anti-miR-25 were designed and synthesized by Shanghai GenePharma (Shanghai, China). The negative control (miR-NC) was also designed by Shanghai GenePharma. The cells were plated in six-well or a 96-well plates (Corning Inc., Corning, NY, USA) before 12 h transfection. Lipofectamine^2000^ Reagent (Invitrogen, Carlsbad, CA, USA) was then used to transfect miRNA or miRNA inhibitor (2′-*O*-methyl modification) into the cells according to the manufacturer’s instruction.

### 4.8. Protein Isolation and Western Blot

Cells were lysed in a Radio Immuno Precipitation Assay Buffer (Pierce, Rockford, IL, USA) with freshly added protease inhibitor cocktail (Roche, Manheim, Germany) for 30 min on ice and were then centrifuged at 16,000× *g* at 4 °C for 10 min. The protein of supernatant concentration was measured with a BCA protein assay kit (Thermo Scientific, Rockford, IL, USA). Whole-cell lysates were separated in 12% SDS-PAGE gels, transferred to PVDF membranes, and then blocked with 5% skim milk for 1 h. The membranes were then incubated with primary antibodies against c-Myc (1:1000, Cell Signaling Technology, Danvers, MA, USA), Cyclin D1 (1:1000, Cell Signaling Technology), DKK3 (1:1000, Cell Signaling Technology) or GAPDH (1:2000, Cell Signaling Technology) at 4 °C for 12 h. After incubation with the primary antibodies, the membranes were washed with TBS/0.05% Tween-20 for three washes and incubated with horseradish peroxidase-conjugated secondary antibodies (1:10,000, Cell Signaling Technology) at room temperature (RT) for 1 h. Signals were detected using enhanced chemiluminescence reagents (Pierce, Rockford, IL, USA). The protein levels were normalized by GAPDH antibody.

### 4.9. 3′-Untranslated Region (3′-UTR) Luciferase Reporter Assays

To generate the 3′-UTR luciferase reporter, the partial sequence of the 3′-UTR from DKK3 was cloned downstream of the firefly luciferase gene in the pGL3-Control Vector (Promega, Madison, WI, USA). Mutation of the miR-25 target sites in the 3′-UTR of DKK3 was used as a control. pRL-TK, which is containing Renilla luciferase, for data normalization. HEK293 cells were seeded in 48-well plates, allowed to adhere overnight, and then transfected using Lipofectamine^2000^ (Invitrogen, Carlsbad, CA, USA). The cells were harvested and assayed with the dual-luciferase assay (Promega, Madison, WI, USA) after two days. Each treatment was tested in triplicate in three independent experiments. The results are expressed as the relative luciferase activity (Firefly LUC/Renilla LUC).

### 4.10. Lentivirus-Based MiR-25 Overexpression

To elucidate the role of miR-25 in vivo, we constructed a recombinant lentivirus termed LV-miR-25 to generate stable gain-of-function miR-25 expression in melanoma cells. The recombinant lentivirus LV-miR-25 and its control LV-miR-NC were prepared as previously described [25].

### 4.11. Tumorigenicity Assays in Nude Mice

BALB/c athymic nude mice (male, five weeks old, 16–20 g) were purchased from Beijing HFK Bioscience Co. (Beijing, China). LTD and bred under pathogen-free conditions. All animal experiments were approved by Xi’an Jiaotong University Medical College Institutional Animal Care and Use Committee. In brief, athymic nude mice were each injected with 5 × 10^6^ LV-miR-253- or LV-miR-CON-infected A375 cells in 150 μL PBS. The tumor volume was measured using the following formula: volume = 0.5 × (length) × (width) × (width). Thereafter, tumor sizes were calculated every other day. When the sizes of the tumors were above the limit described by the animal protocol approved by Xi’an Jiaotong University Medical College Institutional Animal Care, the mice were sacrificed. The tumors of nude mice were removed. Tumors were fixed in 10% formalin (Histochoice Tissue Fixative MB, Amresco, Solon, OH, USA), processed and embedded in paraffin. Immunohistochemistry was performed on 5-μm-thick paraffin sections mounted on charged slides. The slides were stained with hematoxylin and eosin and immunostained with Ki67 to observe proliferation. The sections were also immunostained to detect the level of DKK3.

### 4.12. Statistics

Data were analyzed using the two-tailed Student’s *t*-test. * *p* < 0.05 was considered statistically significant.

## 5. Conclusions

In conclusion, our findings demonstrate that overexpression of miR-25 promotes melanoma cell proliferation and metastasis in vitro and in vivo. miR-25 is activated by the WNT/β-catenin signaling pathway and exerts its pro-metastatic function by directly inhibiting DKK3. Downregulation of miR-25 enhances the induction of c-Myc, TCF4, and Cyclin D1. The miR-25/DKK3 axis provides new insight into the pathogenesis of melanoma, particularly with respect to proliferation and metastasis, and it represents a potential therapeutic target for melanoma.

## Supplementary Materials

Supplementary materials can be found at www.mdpi.com/1422-0067/17/11/1124/s1.
