# Supplementary material for: Upregulated MicroRNA-25 Mediates the Migration of Melanoma Cells by Targeting DKK3 through the WNT/β-Catenin Pathway
Source: Int J Mol Sci. 2016 Oct 27;17(11):1124. doi: 10.3390/ijms17111124 (PMC5133768; doi:10.3390/ijms17111124)
Supplement: Supplementary file 1 [file ijms-17-01124-s001.pdf]

Supplementary Materials: Upregulated MicroRNA-25 Mediates the Migration of Melanoma Cells by Targeting DKK3 through the WNT/β-Catenin Pathway

Jia Huo, Yanfei Zhang, Ruilian Li, Dingwei Zhang and Jiawen Wu

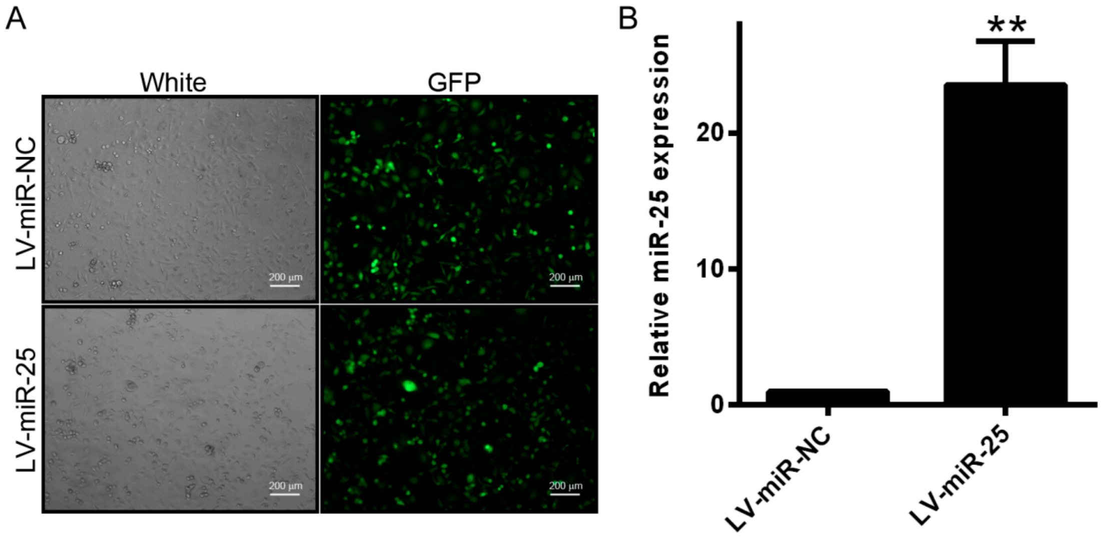

Figure S1. Efficient transfection of lentivirus in A375 cells was shown by using fluorescent microscopy. (**A**) The black and green pictures showed the cells in the same field under normal white light and fluorescence light, respectively. GFP, green fluorescent protein; (**B**) quantitative real-time polymerase chain reaction (qRT–PCR) was used to evaluate relative expression of miR-25 in A375 cells transfected with lentivirus LV-miR-25 or LV-miR-NC. The average miRNA expression in LV-miR-NC group was designated as 1. ** $p < 0.01$.
